# Viral Dynamics in the Tropical Pacific Ocean: A Comparison between Within and Outside a Warm Eddy

**DOI:** 10.3390/v16060937

**Published:** 2024-06-11

**Authors:** Patrichka Wei-Yi Chen, Madeline Olivia, Gwo-Ching Gong, Sen Jan, An-Yi Tsai

**Affiliations:** 1 Institute of Marine Environment and Ecology, National Taiwan Ocean University, Keelung 202-24, Taiwan; 0063b047@email.ntou.edu.tw (P.W.-Y.C.); medelineolivia@gmail.com (M.O.); gcgong@mail.ntou.edu.tw (G.-C.G.); 2 Doctoral Degree Program in Ocean Resource and Environmental Changes, National Taiwan Ocean University, Keelung 202-24, Taiwan; 3 Center of Excellence for the Oceans, National Taiwan Ocean University, Keelung 202-24, Taiwan; 4 Institute of Oceanography, National Taiwan University, Taipei 106319, Taiwan; senjan@ntu.edu.tw

**Keywords:** warm-core eddies, bacteria, viruses, viral–bacterial abundance ratio, downwelling

## Abstract

In mesoscale eddies, the chemical properties and biological composition are different from those in the surrounding water due to their unique physical processes. The mechanism of physical–biological coupling in warm-core eddies is unclear, especially because no studies have examined the effects of environmental factors on bacteria and viruses. The purpose of the present study was to examine the influence of an anticyclonic warm eddy on the relationship between bacterial and viral abundances, as well as viral activity (viral production), at different depths. At the core of the warm eddy, the bacterial abundance (0.48 to 2.82 × 10^5^ cells mL^−1^) fluctuated less than that outside the eddy (1.12 to 7.03 × 10^5^ cells mL^−1^). In particular, there was a four-fold higher viral–bacterial abundance ratio (VBR) estimated within the eddy, below the layer of the deep chlorophyll maximum, than outside the eddy. An anticyclonic warm eddy with downwelling at its center may contribute to viruses being transmitted directly into the deep ocean through adsorption on particulate organic matter while sinking. Overall, our findings provide valuable insights into the interaction between bacterial and viral abundances and their ecological mechanisms within a warm eddy.

## 1. Introduction

Mesoscale eddies occur often in the open ocean because of water mass turbulence [1,2,3], and they include both warm-core eddies (anticyclones) and cold-core eddies (cyclones). A convergence of anticyclonic warm waters in the ocean sinks nutrient-depleted surface waters toward the subsurface, intensifying oligotrophy in the upper euphotic column [1]. The downwelling nature of warm eddies isolates nutrient-depleted water at the surface, presumably supporting the microbial food web by cycling nutrients [4]. According to previous studies, warm eddy analyses have largely been conducted to determine whether physical-chemical variations are a contributing factor to changes in bacterial abundance and activity [5,6,7,8,9,10]. However, there is an apparent gap in the understanding of the top-down control mechanisms affecting bacteria (such as viruses).

In general, viruses affect microbial mortality, as well as the ecological processes and biogeochemical cycles of oceans [11,12]. The lysis of bacteria by viruses disrupts the flow of energy and organic matter by creating a “viral shunt” of bacteria, viruses, and dissolved organic matter [13]. Therefore, viral lysis can contribute to bacterial mortality; on the other hand, viral lysis of bacteria causes the release of cellular materials that provide organic matter for easy uptake by uninfected bacteria [14]. Numerous studies have evaluated the spatial distribution of viruses and their hosts (such as bacteria) in estuaries, coastal waters, and open oceans, as well as in freshwater ecosystems [13,15,16,17,18,19]. It remains unclear how the spatial patterns of bacteria affect viral abundance and activity inside mesoscale eddies.

There is at least one order of magnitude of difference between the abundances of viruses and bacteria in most ecosystems [20]. The viral–bacterial abundance ratio (VBR) is commonly used as an indicator of the relationship between bacteria and viruses. The VBR index determines the severity of virus infection, with high values indicating a high viral dynamic and, consequently, an enhanced lysis of prokaryotic cells [13]. A low value, however, may suggest a decrease in or the absence of viral activity [13,21]. In addition, differences in VBR values are also affected by physicochemical factors (e.g., temperature, salinity, and nutrients) [21]. Generally, viral abundance decreases throughout the photic zone, reaching a constant abundance at depth, and it correlates with the distributions of the most abundant hosts (bacteria and phytoplankton) [13]. Taylor et al. [22] showed that the mean VBR of oxic layers (15) was significantly lower than that of anoxic layers (VBR = 31), suggesting varying relationships among viruses, hosts, and environments. There have been further reports of a high VBR in bathypelagic waters in the open North Atlantic [23] and South Atlantic Oceans [24], as well as in the Pacific Ocean [25]. There is still a lack of understanding of the factors controlling viral dynamics. Even though the VBR is the result of a complex balance of factors that include viral production, virus transport via sinking particles, decay rates, and life strategies [20,26], there is still scarce information on the occurrence and characteristics of viruses and bacteria, especially in the oceanic water column of warm eddies.

To the best of our knowledge, little is known about the viral abundance and viral production response associated with warm eddies from surface to deeper waters. The purpose of the present study was to examine the influence of an anticyclonic warm eddy on the relationship between bacterial and viral abundances, as well as viral activity (viral production), at different depths. To achieve this goal, we conducted field experiments to measure viral production in order to identify the viral dynamics and relationship between bacteria and viruses inside and outside a warm eddy in the tropical Pacific Ocean during warm eddy movement in surface, deep chlorophyll maximum (DCM), 200 m, and 500 m layers. In particular, no studies have investigated lytic viral infection and lysogeny in the contrasting marine environments of warm eddies or both viral life cycles simultaneously. To evaluate the specific role that viruses play in microbial food web processes in different environments, it is necessary to gather such information. Our hypothesis states that there are different viral dynamics (viral abundance and viral production) between warm eddies and the surrounding waters and that they induce different relationships between bacterial and viral abundance.

## 2. Materials and Methods

### 2.1. Study Site and Sampling

**Figure 1:**
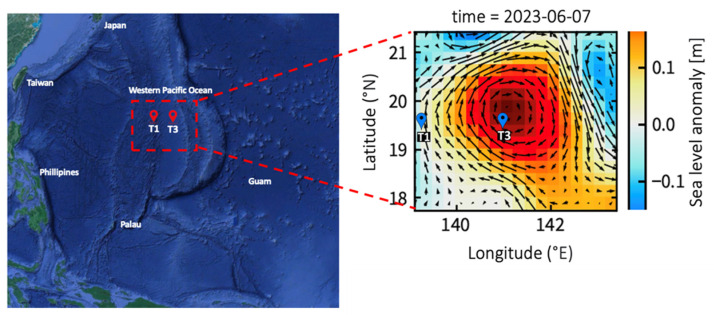
Map showing the sampling stations plotted against the averaged sea surface height anomaly (m) for the study period of 2023. The arrows represent sea surface currents.

An investigation aboard the R/V Thompson was conducted in the West Pacific Ocean from 29 May to 10 June 2023 (Figure 1). During the cruise, the sea surface height was monitored every day using a satellite altimeter (Figure 1). Sea level anomaly (SLA) was calculated from satellite altimeter data using AVISO (Archiving Validation and Interpretation of Satellite Oceanographic Data) to define the warm eddy trajectory (http://www.aviso.oceanobs.com/ 7 June 2023). Several large-scale circulation transport pathways cross the western equatorial Pacific Ocean, connecting the North, South, and Central Pacific Oceans to the tropical Indian Ocean. The North Equatorial Current (NEC), North Equatorial Counter Current (NECC), and North Pacific Subtropical Countercurrent (STCC) form a complex current system. The study area was split into two groups, namely, the eddy core (EC) and out of eddy (OE), based on the closed contour of the sea level anomaly. We monitored the drifting positions of drifting buoys to monitor the same eddy. Teflon-coated Go-Flo bottles were used to collect seawater samples. We used an SBE 9/11plus CTD (Sea-Bird Scientific) to record hydrographic data at various depths, including temperature, salinity, and photosynthetically available radiation (PAR). Furthermore, incubation experiments of viral production at the OE and EC were carried out at a surface depth of 5 m, 130–140 m of the DCM, 200 m, and 500 m. 

### 2.2. Viral Production

For the purpose of estimating viral production from the experiment conducted for 24 h, we used the dilution technique proposed by Wilhelm et al. [27]. Further, lytic viral infections (bacteria at the lytic stage) and lysogeny (bacteria-producing inducible prophages) were examined. The first step in obtaining virus-free water consisted of its filtration through a 0.2 µm pore membrane, followed by tangential flow filtration (TFF) using a molecular weight cut-off of 30 kDa. Then, one-liter samples of prefiltered seawater (2 µm) were concentrated to obtain a volume of 100 mL using a 0.2 µm pore size polycarbonate filter (47 mm diameter, Millipore) under low vacuum (<200 mm Hg), and the concentrated water was transferred using a pipette. Our experiment involved diluting 100 mL of concentrated water with 400 mL of virus-free water. Consequently, the viral density in the seawater was reduced to 20% of what it had been previously, reducing the chances of contact between viruses and hosts, thereby preventing new infections. A chemical agent, mitomycin C (1.0 μg mL^−1^ final concentration; Merck), was used as an inducing agent to switch from a lysogenic cycle to a lytic cycle for the dilution samples collected, while a control dilution sample remained untreated. After that, the diluted samples were incubated in triplicate in 50 mL polycarbonate bottles and a temperature-controlled chamber containing similar in situ temperatures at each depth for 24 h in the dark. During the 24 h incubation period, subsamples of 1 mL were taken in triplicate at 6 h intervals for bacterial and viral enumeration. Briefly, lytic VP could be represented by viral accumulation in control tubes without the addition of mitomycin C. Based on the difference between the mitomycin C-treated and control tubes, lysogenic VP was calculated [28]. 

After correcting for the dilution of bacterial hosts between the samples and the natural communities, the lytic VP and lysogenic VP were calculated by using first-order regressions of viral abundance versus time. This was necessary to account for the loss of potentially infected cells during filtration. They were calculated using the formula proposed by Hewson and Fuhrman [29]: VP_(lytic, lysogenic)_ = m_(lytic, lysogenic)_ × (B/b), where m is the slope of the regression line, b is the concentration of bacteria after dilution, and B is the concentration of bacteria before dilution. In this study, the total VP (TVP) was calculated by adding lytic VP to lysogenic VP. A viral turnover rate (VT, d^−1^) was calculated by dividing TVP by in situ viral abundance. 

We estimated the viral-induced mortality of bacteria (VMB, bacteria mL^−1^ d^−1^) based on an estimate of 24 burst sizes (BS, number of viruses released per lytic event) [30]. As shown in the equation, VMB = TVP/BS. Additionally, the loss rate of bacteria from viral activity (LRB-V) was calculated as VMB/(in situ bacteria). 

The calculation of the bacterial net growth rate (r) was based on the change in abundance during the exponential phase of growth during the study period (24 h). In this study, we used the equation r = (ln N_t_ − ln N_0_)/t, where N_0_ and N_t_ are the bacterial abundances at the beginning of the study, as well as the peak values, and t is the time when bacterial abundance reached its peak. Further, the ratio of bacterial production removed by viral lysis (viral control factor) was estimated using r/(LRB-V).

### 2.3. Flow Cytometric Analyses

Flow cytometry samples of viruses and bacteria were analyzed using a CytoFLEX S flow cytometer (Beckman Coulter, Indianapolis, IN, USA), which was equipped with an argon-ion laser at 488 nm, a 525 nm filter, and an SYBR signal trigger. To reduce particle density interference, the virus samples were diluted 1:10 in TE buffer (pH 8.0, EM grade) before staining. Then, the diluted samples were stained with SYBR Green I (final concentration 1: 50,000 of commercial stock) and incubated in the dark at 80 °C for 10 min. The samples were then cooled in an ice bath to 25 °C and processed through FCM in accordance with Brussaard [31]. We performed blank controls using TE buffer stained with the same concentration of SYBR Green I to detect and eliminate buffer noise. Before FCM processing, the bacteria samples were stained for 15 min with SYBR Green I at a concentration of 1:10,000, as described by Hammes and Egli [32]. Based on flow cytometric analysis, on the basis of their red fluorescence from chlorophyll (>650 nm) and light scatter signals (SSC), *Prochlorococcus* spp. were counted according to Calvo-Díaz and Morán [33].

### 2.4. Statistical Analysis

Before analysis, Shapiro–Wilk W tests were conducted to ensure that the data were normal, and the data were logarithmically transformed to achieve normality when necessary. A linear regression analysis was performed to analyze the relationship between viral abundance and the time of triplicate incubation. The regression lines were tested using an analysis of variance (ANOVA). Moreover, the significance between the slopes of the control and mitomycin C treatment was determined using an *F*-test. If the regression slopes of the control and mitomycin C treatment were significantly different, we calculated the magnitude of lysogenic VP. Conditions inside and outside of the eddy were compared and analyzed using *t*-test. STATISTICA 7.0 software was used for all statistical operations. A probability value of <0.05 was considered significant. Means and standard deviations are used whenever necessary to express the results.

## 3. Results

### 3.1. Station Characteristics

**Figure 2:**
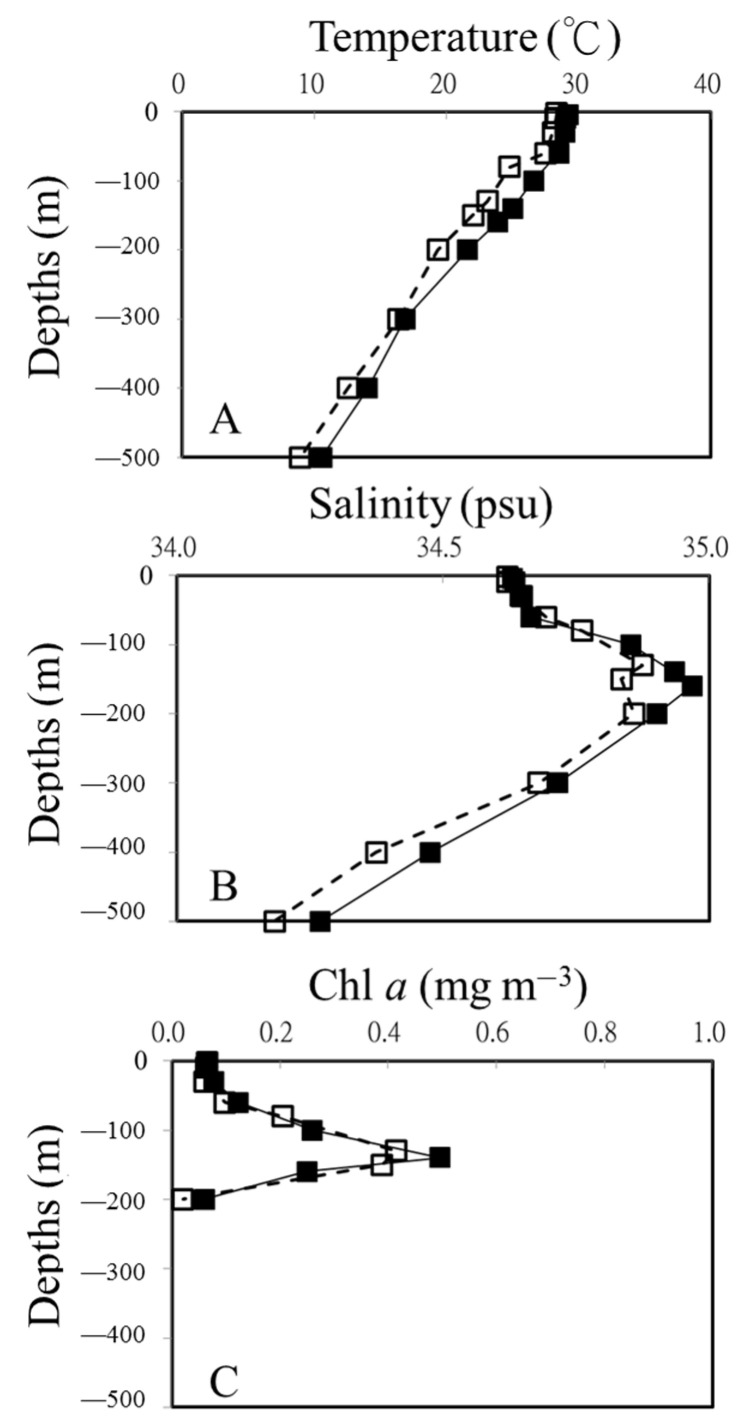
Vertical profiles of temperature (**A**), salinity (**B**), and Chl *a* (**C**) at out of eddy (OE) (white squares) and eddy core (EC) (black squares).

The warm eddy had a significant effect on the vertical distributions of temperature and salinity in the study area (Figure 2). Based on the temperature and salinity profiles of the OE and EC, a well-mixed surface layer reached a depth of ca. 50 m (Figure 2A,B). The surface water temperature was at least 1 °C higher at the eddy core (EC) than in the surrounding water (OE). As shown in Figure 2A,B, in the upper 500 m depth of the EC, the water temperatures ranged from 10.6 to 29.2 °C, and salinity varied from 34.27 to 34.97 psu. Compared with the sampling points in the same layers in the OE, both temperature and salinity were higher in the warm eddy (Figure 2). A dome-like distribution of cold and low-salinity water was observed at 15–20° N at the junction of the NEC and the STCC (Figure 2B). The chlorophyll a concentration in the surface water was similar inside and outside the eddy. A deep chlorophyll maximum (DCM) at the OE was centered at about 130 m, whereas a deeper DCM was found within the eddy (Figure 2C).

### 3.2. Bacterial and Viral Abundance

**Figure 3:**
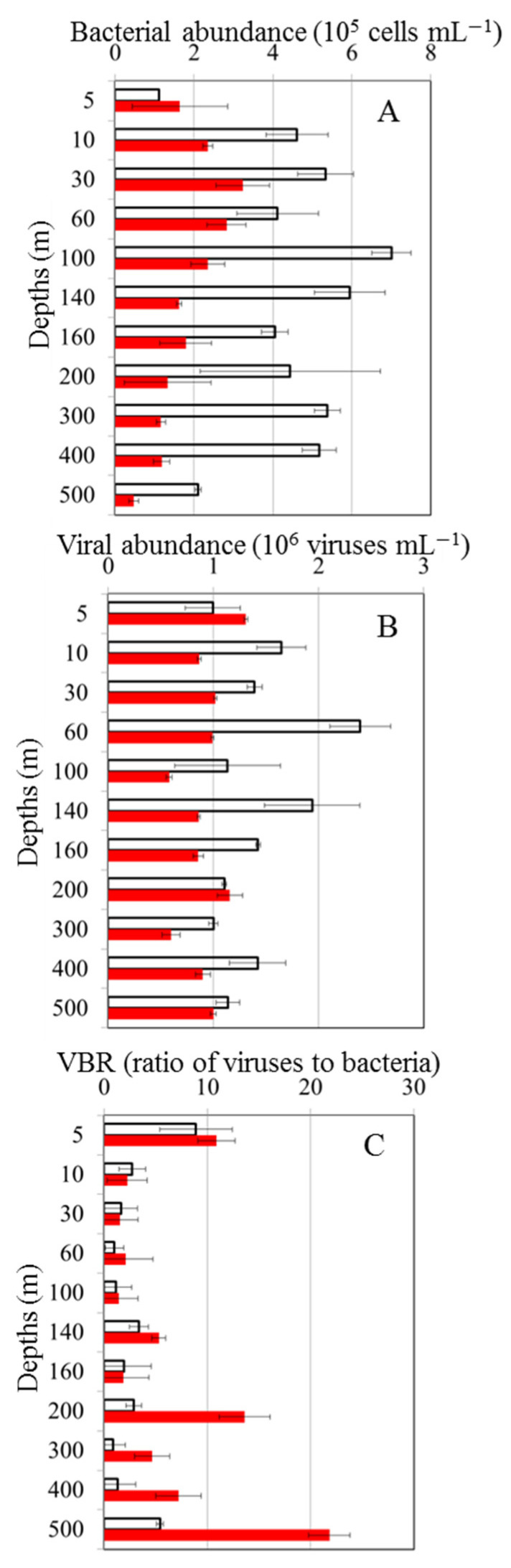
Vertical variations in mean and standard deviation of bacterial abundance (**A**), viral abundance (**B**), and virus-to-bacteria ratio (VBR) (**C**) at out of eddy (OE) (white bars) and eddy core (EC) (red bars).

The abundance of bacteria at the OE station ranged from 1.12 to 7.03 × 10^5^ cells mL^−1^ from the surface to the 500 m depth, with the lowest abundance observed at the surface and the highest abundance observed at a 100 m depth (Figure 3A). At the EC station, bacterial abundance (0.48 to 2.82 × 10^5^ cells mL^−1^) fluctuated less than that at the OE station (Figure 3A). Viral abundance was greater than bacterial abundance, and it was 0.81 × 10^6^–2.60 × 10^6^ viruses mL^−1^ and 0.60 × 10^6^–1.31 × 10^6^ viruses mL^−1^ at the OE and EC stations, respectively (Figure 3B). There were significant differences in the bacterial and viral abundances inside and outside of the eddy (*t*-test, *p* < 0.05), with higher abundances of bacteria and viruses observed at the OE station (Figure 3A,B). Furthermore, a large variation in the VBR was observed at the EC station with depth, and the values differed significantly between the two stations from the 200 m to 500 m depths (*t*-test, *p* < 0.05), with higher VBR values in the EC region (Figure 3C).

### 3.3. Viral Production

**Figure 4:**
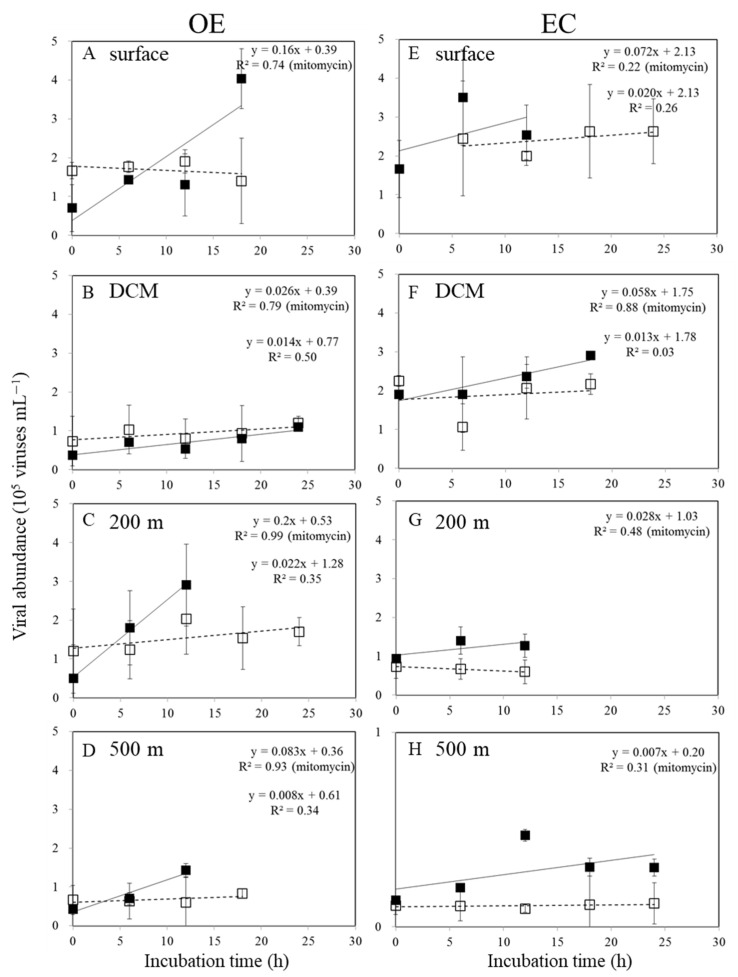
Temporal variations in mean and standard deviation of viral abundance during 24 h incubations for viral production in the surface water (**A**,**E**), DCM (**B**,**F**), 200 m (**C**,**G**), and 500 m depth (**D**,**H**) at OE and EC stations. □: control treatments; ■: mitomycin treatments.

Viral abundances varied after viral reduction and during the 24-h incubation experiment in the control and mitomycin C-treated samples, as expected from inducible lysogenic bacteria (Figure 4). We analyzed the data to determine viral production and found that lytic and lysogenic bacteria were primarily detected after 12–24 h (Figure 4). As shown in Figure 4 and Table 1, the heterotrophic bacterial community displayed very high levels of lysogeny (100%) at the surface and below the DCM in the OE and EC regions, respectively. According to prophage induction due to mitomycin C, lysogenic production was found to range from 72.3–100% in the bacteria communities in the EC region, with the highest values (100%) found in deep waters (200 m and 500 m) (Table 1). At the OE and EC stations, the total virus population (TVP) varied widely, with values of 0.63~4.80 and 0.17~1.73 × 10^5^ viruses mL^−1^ h^−1^, respectively (Table 1). The viral turnover rate (VT) ranged from 0.03 to 0.43 d^−1^ and from 0.02 to 0.16 d^−1^ at the OE and EC stations, respectively (Table 1). The TVP and VT were significantly higher at experimental depths at the OE station than at the EC station, except for in the DCM layer (Table 1).

### 3.4. Virus-Mediated Bacterial Mortality

Assuming a BS of 24 (see Section 2.2), the estimates of the virus-mediated loss rate of bacteria (VMB) varied between 2504 and 19,200 cells mL^−1^ d^−1^ at the OE station and between 680 and 6920 cells mL^−1^ d^−1^ at the EC station (Table 1). As with the experimental depths, the VMB decreased markedly with increasing depths at the EC station, but the lowest value (2504 cells mL^−1^ d^−1^) was found in the DCM layer in the OE region (Table 1). This implies that, on average, from 0.004 to 0.14 d^−1^ of the bacterial standing stock was removed daily due to viral lysis (LRB-V) in the OE region. The estimates of LRB-V ranged between 0.01 and 0.04 d^−1^ and displayed small spatial patterns at the EC station (Table 1). In comparison with bacterial growth, the ratio of LRB-V to bacterial growth rate (viral control factor) was higher (0.86~0.99) in the OE region, except for in the DCM layer (Table 1). At the EC station, the values of the viral control factor (0.25~0.35) were significantly lower than those at the OE station (Table 1).

## 4. Discussion

In the ocean, eddies are energetic, swirling, and time-dependent circulations that are approximately 100 km wide, and they can be either warm-core (anticyclone) or cold-core (cyclone) eddies. A satellite-derived dataset allows researchers to study eddies in large spatiotemporal domains and over high-frequency intervals and determine their biological functions. Recently, there have been many observations of mesoscale eddies in the tropical Pacific Ocean [34,35], but little is known about their role in the bacterial and viral dynamics there. The mechanism of physical–biological coupling in warm-core eddies is unclear, especially because no studies have examined the effects of environmental factors on bacteria and viruses.

### 4.1. Vertical Patterns of Bacterial Abundances

Interestingly, the OE and EC stations showed different vertical patterns of bacterial abundance, with the abundance being lower in the EC station than in the OE station (Figure 3A). In different aquatic environments, different factors may limit bacterial growth [36,37]. The effects of dissolved organic matter (DOM), inorganic nutrients, and temperature on bacterial growth were studied in previous studies [38,39]. A tropical marine ecosystem has warm surface temperatures year-round, as shown in this study. At high temperatures, there is less new nutrient input in the open ocean because of stratification [40]. It may be that tropical heterotrophic bacteria are less responsive to temperature since DOM supply plays a strong role in bottom-up control. In a study by Shiah and Ducklow [41], it was reported that the growth rate of bacteria is limited by a temperature below 20 °C in the absence of a substrate limitation. There is also evidence from previous studies showing that DOM affects bacterial production in the equatorial Pacific Ocean [38]. Even though temperature played a major role in explaining the variation in bacterial properties at other study sites, it is unlikely that spatial variability could be explained by temperature because the temperature difference between the outside and inside of the warm eddy never exceeded 3 °C at the sampling depths.

The regeneration of carbon and nutrients is particularly important in an oligotrophic aquatic environment with a low DOM and inorganic nutrients, as it is required to enhance bacterial growth [41,42]. With increasing emphasis on carbon and nutrient regeneration in marine systems in the last decade, a better understanding of planktonic viruses has become essential. The process of nutrient regeneration in microbial communities remains difficult to measure directly. Some studies have shown, however, that virally mediated nutrient recycling plays an important role in some systems, including in laboratory experiments with viral–host systems [41], as well as in field studies [43]. Our results show that the total viral production (TVP) was higher in the OE region (0.46~4.8 × 10^5^ viruses mL^−1^ h^−1^), with higher regeneration rates of carbon and other nutrients supporting bacterial growth, but the overall bacterial growth rates at the OE station were not higher than those at the EC station (Table 1). We inferred that higher viral production led to greater regeneration rates of carbon and other nutrients, but we did not observe higher bacterial growth rates in the OE region (Table 1). There is also a possibility that variations in bacterial mortality may explain the lower bacterial abundance at the EC at sampling depths. According to some studies, higher temperatures increase grazing pressure in anticyclonic eddies [3]. Similarly, Boras et al. [44] found high grazing activity at stations subjected to anticyclonic eddies, which might be associated with a higher abundance of nanoflagellates. An alternative explanation is that anticyclonic eddies have a convergence effect that drives microzooplankton and nanoflagellates toward the core of the eddy. This can result in increased microzooplankton densities in surface waters, thus increasing the bacterial grazing rate [45].

### 4.2. Percentage of Lysogenic Viral Production

Subsamples of the incubation bottles were collected from the experimental sample to determine the viral production rate. A precise sampling time is critical here since the rate of virus production within samples can be determined by this information. During times of rapid microbial turnover, this may occur every 1.5 h, whereas during times of slow microbial growth, it may occur every 4–6 h [46]. In the present study, we collected subsamples at 6-h intervals because of the low growth rates of the bacteria (Table 1). The prevalence of lysogenic viral infection varied significantly over the different depths outside and inside the warm eddy (Table 1). Bacteriophage reproduction mostly occurs through lytic or lysogenic infections, and the quantitative significance of these processes varies across different oceans [11]. In previous studies, lytic and lysogenic viral infections in seawater were found to be associated with the trophic status of the marine systems [26]. It is thought that productive systems favor the lytic cycle, which is dependent on frequent virus contacts, while low-abundance hosts may allow for lysogenic infection [44]. In this study, we obtained new data on a variety of viral parameters in the tropical Pacific Ocean, with a particular focus on warm eddy regions. As shown in Table 1, the percentage of lysogenic cells outside the warm eddies could reach 100% in the surface layer. We found a lower abundance at these depths (1.1 × 10^5^ cells mL^−1^) and, similar to other studies, evidence indicating that lysogenic infections are the most effective under unfavorable conditions, particularly in waters that are low in bacteria and primary production [47,48]. Additionally, lysogeny can contribute to the resistance of a host to stressors by expressing advantageous genes carried by the virus [48]. Thus, the virus can protect the host against stressors, such as UV radiation [49]. In this situation, another explanation for the high percentage of lysogenic cells in the surface layer in the OE region could be the high UV radiation in the tropical ocean. Furthermore, a high percentage of lysogenic production was also observed at both stations at a 500 m depth (Table 1). It is worth mentioning that viruses are resistant to low temperatures, as they change their lytic life strategy into a lysogenic one when environmental conditions are unfavorable for prokaryotic growth [50].

### 4.3. Virus-to-Bacteria Ratios

**Figure 5:**
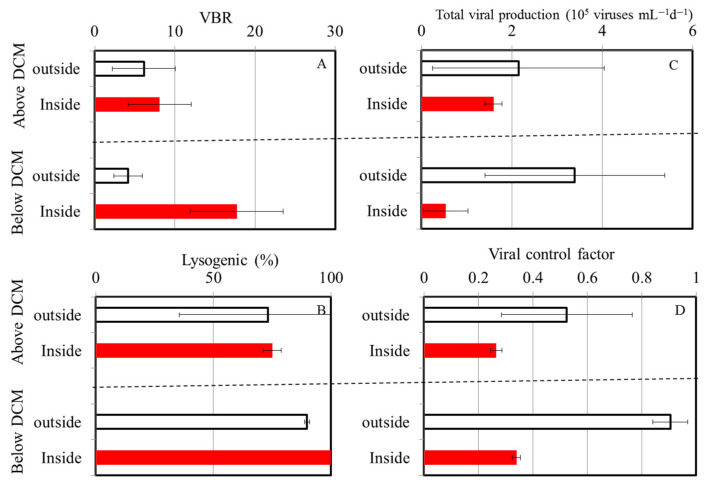
Mean and standard deviation of virus-to-bacteria ratio (VBR) (**A**), the percentage of lysogenic to total viral production (%) (**B**), total viral production (**C**), and viral control factor (**D**) above and below DCM region at OE (white bars) and EC (red bars).

Furthermore, we compared the surrounding and inner water columns of the warm eddy, which could be divided into two layers, above and below the DCM depth (Figure 5). Using these analyses, we found no significant difference in the virus-to-bacteria ratios above the DCM at both stations, with a mean of 6.1 ± 3.9 and 8.1 ± 3.9 at the OE and EC stations, respectively (Figure 5A). However, the values estimated for the EC region (17.7 ± 5.8) below the DCM were four-fold higher than those estimated for the OE station (4.2 ± 1.8) (Figure 5A). Another observation suggests that temperature can influence changes in the VBR in the water column; decreasing temperatures were associated with a greater survival of viruses [49]. Due to this, a decrease in temperature of 8~10 °C in the mesopelagic waters of the tropical Pacific Ocean is likely to lead to increased virus survival rates. In this study, it is unlikely that temperature accounted for the difference in the VBR because of the small temperature difference between the inside and outside of the warm eddy below the DCM. 

To study the relationships between viral and bacterial communities, the VBR has been widely used [11,15,21]. The VBR determines how viruses infect hosts, i.e., high values indicate high viral dynamics and, as a consequence, a high rate of bacterial cell lysis [11]. Alternatively, low values are interpreted as diminishing or absent viral activity [11,15,21]. The viral production measured below the DCM in the EC region in our study, however, did not resemble these previous observations, with a lower viral production and bacterial control factor below the DCM being observed (Figure 5C,D). In the present study, low viral production did not result in a higher viral abundance below the DCM at the EC and, consequently, a higher VBR. Taking into account the variations in the factors in Figure 5, we concluded that the differences between the EC and OE in the VBR below the DCM region may have more to do with factors other than temperature and viral production.

**Figure 6:**
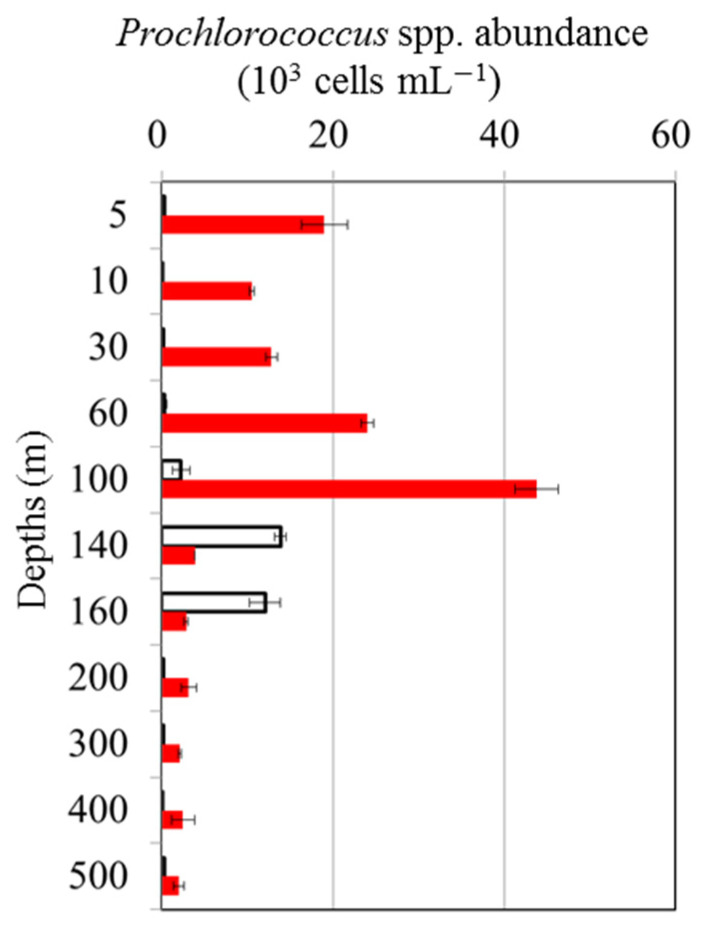
Vertical variations in mean and standard deviation of *Prochlorococcus* spp. abundance at OE (white bars) and EC (red bars).

Particle vertical sinking is one of the underlying mechanisms driving oceanic dynamics at a global scale in physical, chemical, and biological processes. With regard to sinking, a previous study suggests that vertical sinking may enhance both lytic and lysogenic production through the promotion of burst size, which is likely to be caused by an increased supply of nutrients, and that it may trigger a switch from a lysogenic to a lytic strategy, probably as a result of environmental changes [51]. According to our study, this pattern differs from that in the previous study by Wei et al. [8] because there was no significant difference in lysogenic viral production between the regions above and below the DCM at either station (Figure 5B). Aside from this, viruses can also be transmitted into the deep ocean indirectly through infected cell hosts or directly through adsorption on particulate organic matter during sinking [52]. However, the interaction between bacteria and viruses and their role in these processes remains largely unknown. To the best of our knowledge, this is the first report regarding the response of bacteria and viruses to the prevailing physical forces associated with an anticyclonic warm eddy in the tropical Pacific Ocean. In general, warm-core eddies are clockwise circulations that raise the sea level, usually with downwelling at their centers. Previous research suggests that the convergence effect drives microbial communities toward anticyclonic eddies [46]. Meanwhile, warm eddy cores also contain an increased amount of particulate organic matter due to this physical function. In different oceanographic regimes, warm-core eddies lead to varying biological responses. Combining previous discoveries with the physical mechanisms of warm eddies, we propose that the downwelling in warm eddies enhances the sinking of particles to the deeper layers. It is also possible for viruses to be transmitted into the deep ocean directly via adsorption on particulate organic matter when the particles sink. In addition, we also observed that the abundance of *Prochlorococcus* spp. in the EC samples was higher than that in the OE samples at 200~500 m (Figure 6). The light irradiance was insufficient for phytoplankton below 200 m. Considering that there were few differences in light at the EE and EC stations, the downward sinking of picophytoplankton from the upper waters to deeper waters led to the enhancement of picophytoplankton at the EC station at 200 m~500 m. A slight chance of warm eddies increasing the VBR is possible because viruses sink downward from the surface with the sinking process.

Due to the unique physical processes of eddies, the chemical properties and biological composition of the water within and surrounding an anticyclonic eddy are significantly different. This study period consisted of two field investigations (within and outside an eddy) designed to demonstrate how a warm eddy affects bacterial and viral abundance distributions. There were significant differences in the bacterial and viral abundances inside and outside of the eddy, with a higher abundance of bacteria and viruses being observed outside of the eddy. In anticyclonic eddies, there appeared to be high grazing activity on bacteria, which may have controlled bacterial abundance. In particular, four-fold higher VBR values were estimated within the eddy below the DCM than outside the eddy. Overall, our findings provide valuable insights into the interaction between bacterial and viral communities and their ecological mechanisms within a warm eddy. 

## Figures and Tables

**Table 1:** Mean and standard deviation of temperature, salinity, bacterial abundance, viral abundance, virus-to-bacteria ratio (VBR), and viral activity (lytic production, lysogenic production, lysogenic (%), total viral production (TVP), viral turnover rate (VT), viral-induced mortality of bacteria (VMB), loss rate of bacteria from viral activity (LRB-V), and viral control factor) and bacterial growth rates at the sampling stations and depths. #: 24 burst sizes (BS, number of viruses released per lytic event). * The regression slope between control and mitomycin C treatment was significantly different (*p* < 0.05).

		Temperature	Salinity	Virusrs	Bacteria	VBR	Lytic Production	Lysogenic Production	Lysogenic (%)	TVP	VT	VMB #	LRB-V	Bacterial Growth Rate	Viral Control Factor
		(℃)	(psu)	(10^6^ viruses mL^−1^)	(10^5^ cells mL^−1^)		(10^5^ viruses mL^−1^d^−1^)	(10^5^ viruses mL^−1^d^−1^)		(10^5^ viruses mL^−1^d^−1^)	(d^−1^)	(cells mL^−1^d^−1^)	(d^−1^)	(d^−1^)	
T1	surface	28.33	34.62	1± 0.26	1.12 ± 0.01	8.90 ± 2.43	0.00	3.84 *	100.0	3.84	0.38	15,360	0.14	0.14	0.99
	DCM	22.00	34.84	1.94 ± 0.45	5.95 ± 0.85	3.36 ± 1.27	0.34	0.29	46.3	0.63	0.03	2504	0.004	0.07	0.06
	200 m	19.33	34.86	1.11 ± 0.02	4.44 ± 2.11	2.89 ± 1.54	0.53	4.27 *	89.0	4.80	0.43	19,200	0.04	0.05	0.86
	500 m	8.95	34.18	1.14 ± 0.11	2.1 ± 0.08	5.41 ± 0.22	0.19	1.80 *	90.5	1.99	0.17	7960	0.04	0.04	0.95
T3	surface	29.19	34.63	1.31 ± 0.01	1.65 ± 1.20	10.87 ± 3.24	0.48	1.25	72.3	1.73	0.13	6920	0.04	0.17	0.25
	DCM	25.00	34.93	0.86 ± 0.01	1.63 ± 0.06	5.32 ± 0.29	0.31	1.08	77.7	1.39	0.16	5560	0.03	0.12	0.28
	200 m	21.59	34.90	1.16 ± 0.08	1.34 ± 1.09	13.61 ± 4.24	0.00	0.67 *	100.0	0.67	0.06	2688	0.02	0.06	0.33
	500 m	10.57	34.27	1 ± 0.03	0.48 ± 0.12	21.83 ± 5.11	0.00	0.17 *	100.0	0.17	0.02	680	0.01	0.04	0.35

## Data Availability

All data are provided in the main text.
